# Enhanced detection of viruses for improved water safety

**DOI:** 10.1038/s41598-023-44528-2

**Published:** 2023-10-13

**Authors:** Emalie K. Hayes, Madison T. Gouthro, Megan Fuller, David J. Redden, Graham A. Gagnon

**Affiliations:** Centre for Water Resources Studies, Department of Civil and Resource Engineering, Dalhousie University, 1360 Barrington Street, Halifax, NS B3H 4R2 Canada

**Keywords:** Civil engineering, Environmental impact

## Abstract

Human viruses pose a significant health risk in freshwater environments, but current monitoring methods are inadequate for detecting viral presence efficiently. We evaluated a novel passive in-situ concentration method using granular activated carbon (GAC). This study detected and quantified eight enteric and non-enteric, pathogenic viruses in a freshwater recreational lake in paired grab and GAC passive samples. The results found that GAC passive sampling had a higher detection rate for all viruses compared to grab samples, with adenovirus found to be the most prevalent virus, followed by respiratory syncytial virus, norovirus, enterovirus, influenza A, SARS-CoV-2, and rotavirus. GAC in-situ concentration allowed for the capture and recovery of viral gene copy targets that ranged from one to three orders of magnitude higher than conventional ex-situ concentration methods used in viral monitoring. This simple and affordable sampling method may have far-reaching implications for reducing barriers associated with viral monitoring across various environmental contexts.

## Introduction

Human pathogenic viruses are responsible for a substantial portion of human morbidity and mortality^1,2^. Woolhouse and Gaunt (2007) found that two-thirds of the 87 novel pathogens first detected between 1980 and 2005 were due to viruses^3,4^. Viral prevalence in freshwater environments is a known path for human pathogen transport and infection due to public exposure through recreational activities and drinking water sources^5–7^. Recreational waters and drinking water sources are susceptible to contamination by pathogenic viruses from multiple pathways^8^, most commonly through undertreated or untreated wastewater discharge and surface runoff into receiving bodies containing human and animal fecal matter^9–11^. Viral presence and persistence in wastewater also suggest potential hazards linked to its agricultural reuse. Using contaminated wastewater or freshwater to irrigate crops could indirectly transmit viruses through product handling or consumption^12^. Most research to date has focused on the detection of enteric viruses in freshwater sources, as these viruses are known to replicate in the gastrointestinal tract of infected hosts, shed in high volumes in fecal matter for prolonged durations, and be transmitted through fecal–oral exposure pathways in contaminated water^5,6^. However, recent work has revealed that several respiratory viruses are also shed through the gastrointestinal tract. Viruses can persist and remain viable in water sources for several days to weeks, depending on water quality characteristics and environmental conditions^13–15^. This is a significant development, as the majority of emerging human viruses, upwards of 85%, are known to be non-enteric RNA-stranded viruses, i.e. severe acute respiratory syndrome coronavirus 2 (SARS-CoV-2) and Influenza A and B (INFA/INFA)^3,4^. The COVID-19 pandemic illustrated the importance of non-enteric viral tracking in aqueous environments, particularly wastewater effluents, to understand respiratory viruses' fate, transport, and infectivity in environmental reservoirs^12,16^. There have been significant advancements in detecting and identifying viruses in the environment through molecular-based methods, such as quantitative polymerase chain reaction (qPCR), which can be used to evaluate the presence of genes of interest (i.e., viral or indicator genes). However, despite improvements in viral detection methods, the occurrence, abundance, and persistence of enteric viruses in freshwaters remain understudied. In the case of non-enteric viruses, they are nearly entirely uninvestigated due to the lack of routine monitoring. Virus monitoring in freshwaters remains challenging due to the inefficient, time-intensive, and costly methods currently available to recover and concentrate viruses from aqueous environments^17^.

There is a need for rapid, simple, and cost-effective monitoring of established and emerging viruses, both enteric and non-enteric, in freshwaters to improve public health protection and anticipate possible future pandemic threats^4,8,18,19^. Due to the health risks of pathogenic viruses in freshwater, many jurisdictions have established drinking and recreational water microbial guidelines to protect the public from infection. Water quality guidance recommends using fecal indicator organisms (FIOs) to characterize fecal contamination and, by extension, viral presence in freshwaters^17,20–22^. For context, Canadian drinking water guidelines recommend treatment to a 4-Log reduction standard for enteric viruses in both surface and groundwater sources^20,21^. Further, Canadian guidelines recommend that routine microbiological monitoring in treated drinking water be limited to Total coliforms and *Escherichia coli* (*E. coli*) without standard monitoring for individual viruses^18^. Recreational water quality guidance in Canada, and more broadly, is also limited to monitoring *E. coli* and enterococci^21^. However, these FIOs are known to be poor surrogates for viral abundance, with weak correlations reported between enteric viruses and FIOs, such as enterococci and *E. coli*^23–25^. FIOs also offer no details on viral specificity, which depends on the disease burden of the population producing the wastewater contribution, differing rates of inactivation during treatment, and environmental degradation processes specific to different viruses^21,26–29^. While FIOs can indicate potential risk of exposure to pathogens in freshwater environments, they are not reliable for monitoring viral presence or exposure risk to viruses in freshwater environments. This is problematic for source water management and drinking water treatment approaches because different viruses have varying survival and inactivation susceptibilities and require different levels of disinfection^28,30^.

Viral monitoring in freshwater is challenging because of the low concentrations of viruses distributed heterogeneously in large bodies of water^8,19,31^. While these low concentrations are difficult to monitor, they are significant enough to cause human disease^32,33^. To ensure accurate results, robust sampling methods typically involve collecting and concentrating large volumes of water, ranging from 10 L to over 1500 L. Current guidance and methodologies recommend filtering at least “a few hundred liters” of surface water sources intended for drinking water, at least 1500 L of groundwater, and up to 1000 L of recreational water for virus concentration^17,22,34^. Various ex-situ concentration methods have been developed to concentrate trace amounts of viruses from large volumes of water, but are often time intensive and cumbersome^17,19,35^. Although downstream processing and analysis are critical components in the overall detection process, the initial water sampling technique for virus monitoring in freshwater environments frequently serves as a limiting factor, particularly in settings with constrained resources. Bofill-Mas and Rusinol^19^ reviewed 59 research articles for viral concentration procedures and found precipitation/flocculation, centrifugation, and filtration (ultra-, electronegative, and electropositive) to be the dominant processes used in recent research. However, these concentrations require a substantial volume of water for analysis and a significant amount of time and energy for concentration. For example, Schijven et al. 2019 developed a Quantitative Microbiological Risk Assessment process for the evaluation of the health risk of adenovirus in drinking water, which required the collection of thirty-five water samples, each approximately 600 L, that was passed through an ultrafiltration unit before elution of the filters for preparation for qPCR^36^. Because of the laborious and impractical methods available for virus concentration, viral detection in drinking and recreational waters is costly and rarely done.

The need for rapid, affordable, and simple viral monitoring to understand the spread of SARS-CoV-2 has led to the refinement of passive sampling techniques initially developed in 1948 to monitor poliovirus in drinking water sources^37^. Passive sampling is an in-situ concentration method which deploys adsorbents to concentrate target analyte based on diffusion-driven adsorption/sorption processes^38,39^. The recent pandemic response led to the evaluation of several different adsorbent media for capturing a range of viral targets from water and wastewater matrices. Hayes et al. (2022, 2023) utilized granular activated carbon (GAC) to effectively capture and recover SARS-CoV-2, INFA, respiratory syncytial virus (RSV), measles (MeV), pepper mild mottle virus (PMMoV), and CrAssphage from wastewater^38,40^. Compared to other adsorbents, enhanced sensitivity and reproducibility was demonstrated when using GAC. This passive approach is advantageous as it allows for prolonged deployment in an aqueous environment, resulting in time-integrated measurements. This work aims to evaluate the effectiveness of passive sampling in capturing and concentrating other viruses of concern in freshwater environments, building on the advancements made in SARS-CoV-2 sampling methods.

This research utilizes established qPCR techniques, coupled with a novel GAC passive sampling program to evaluate the presence of both enteric and non-enteric viruses in freshwater. Viruses can adsorb to particles rather than remain detached and free-floating in water^41,42^. The application of passive sampling in this context provides in-situ particulate concentration in the water column by capturing small fractions of suspended or settling particles and adsorbing free-floating viruses if present^39,43^. Others^42,43^ have shown that activated carbon can readily adsorb enteric viruses and coliphages, including adenovirus (AdV), rotavirus (RV), norovirus (NV), and bacteriophage MS2, from a range of source waters. While much of this research has focused on activated carbon for point-of-use water treatment for virus sequestration, this current study seeks to exploit the adsorptive nature of activated carbon as an in-situ concentration technique for viral monitoring. This work aims to investigate a novel GAC-based passive sampling technique for viral detection in a freshwater recreational lake. Passive samplers were deployed to detect the presence of 8 common enteric and non-enteric pathogenic viruses, including enterovirus (EnV), AdV, NS, RV, INFA, RSV, SARS-CoV-2, and MeV through in-situ concentration to address the challenges of virus capture and concentration in freshwater.

## Results and discussion

### Prevalence of human viruses in freshwater environments

Across three months, 20 passive sampling events and 33 grab sample events occurred at two locations in a freshwater lake in Nova Scotia, Canada. Grab and passive samples were analyzed by RT-qPCR methods to determine the presence of SARS-CoV-2, Influenza A, RSV, Measles, Adenovirus, Enterovirus, Rotavirus, and Norovirus. The general water quality of the lake is shown in Table S1; briefly, the water temperature in the lake ranged from 14.1 to 25.5 °C, dissolved organic carbon (DOC) ranged from 2.2 to 2.5 mg L^−1^, and pH ranged from 5.7 to 7.9. Importantly, these water quality parameters would indicate a healthy lake within the region and did not exhibit any signs of significant water quality inconsistencies or contamination.

**Figure 1:**
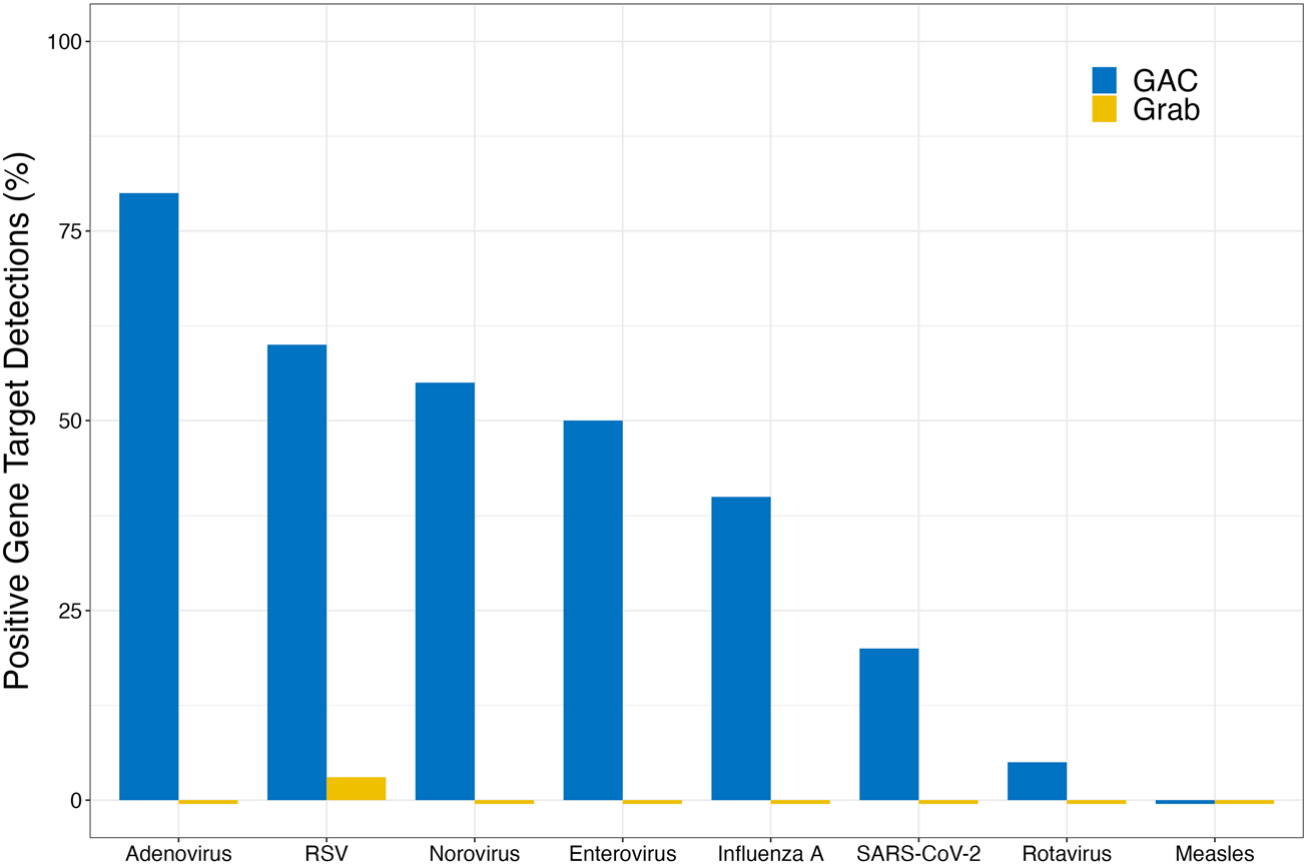
Percent positive gene target detections for the eight viruses using the two sampling methods. The detection frequencies were computed using the total number of samples from both locations.

Grab samples were found to have a 0.38% detection rate for viruses included in this study. There was a single grab sample detection for RSV in June of the study period. GAC passive samples were found to have a 38.8% total positive detection rate, with seven of the eight viruses included in this study being detected. No MeV was present in the passive samplers, which was expected given that Canada has no-sustained circulation of this virus^44^. As shown in Fig. 1, AdV was the most prevalent, with an overall positive detection of 80%, followed by RSV, NV, EnV, INFA and SARS-CoV-2, each with positive detections of 60%, 55%, 50%, 40% and 20%, respectively. RV was detected at the lowest prevalence with a positive detection of 5%. These detection frequencies are particularly notable in the study lake because there is no centralized municipal wastewater effluent inflow to the lake. Human viral inputs are limited to direct human vectors during recreation, overland runoff during rain events, subsurface discharge from nearby septic systems, or possible unregulated direct discharge from shoreline residences.

The findings presented in Fig. 1 indicate the positive detection rates for the two sampling locations. Statistical analysis found no significant differences in concentrations of the target genes between the two locations (*p* < 0.05, Wilcoxon signed rank test), except for the RSV target. At Site 1, the RSV gene concentrations were detected at approximately 1.3 × 10^7^ GC (CI = 6.4 × 10^5^ to 5.7 × 10^8^ GC) more than at Site 2 (*p* = 0.03). The cause for this spatial variation for RSV is unknown, as RSV has not been previously studied in freshwater.

The absence of viral detection in relatively small volumes of freshwater highlights why most recreational water guidance relies on *E. coli* and enterococci monitoring to estimate fecal contamination. Monitoring of FIOs in this study found *E. coli* and enterococci concentrations to have a geometric mean of 14.9 CFU per 100 mL and 46.1 CFU per 100 mL, respectively. The low *E. coli* concentrations and undetectable viral concentrations in grab water samples indicate mild fecal contamination and limited viral presence. However, passive sampling revealed high viral prevalence in the same water body, highlighting the uncertainty of using grab samples alone for monitoring viral occurrence in freshwaters. Grab samples may not always reflect the actual microbial load due to spatial and temporal variations in microbial distribution. Results indicate that passive samplers provide an effective in-situ concentration of otherwise undetectable levels of viral presence. However, meaningful interpretation of this data will require methods for correlating the amount of virus accumulated in the sampler with the corresponding human health impact.

**Figure 2:**
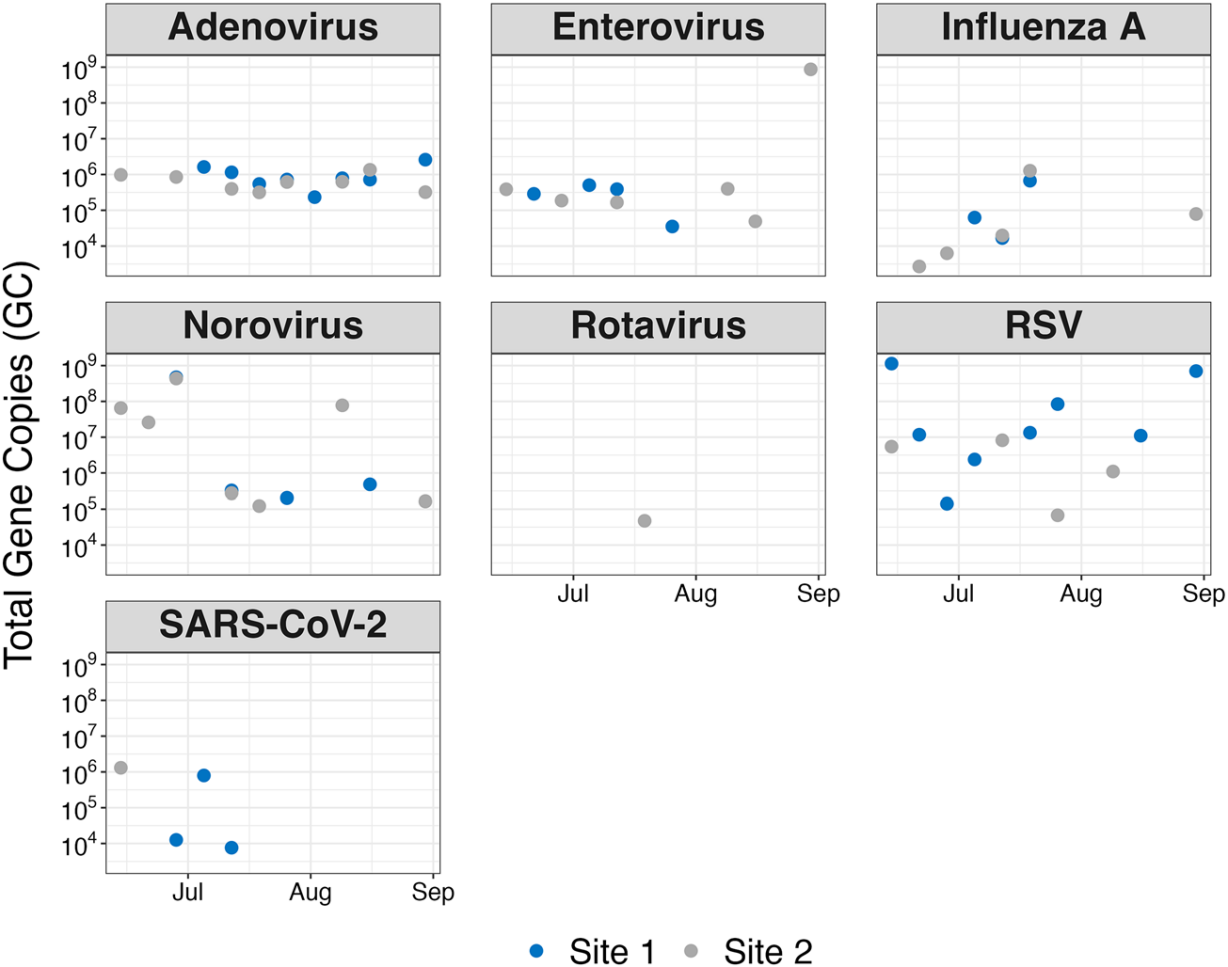
Concentrations of total viral gene copies detected from the GAC passive sampler. The concentrations obtained from each of the two sample locations are shown and denoted by the points' colour. Samplers at both sampling sites were deployed and collected weekly. MeV is not shown, as it was not detected in any samples.

The total number of gene copies detected for each virus per sampling event is shown in Fig. 2. The maximum total gene copies recovered during the sampling period for each virus were as follows: INFA (4.2 × 10^5^ GC), RSV (3.9 × 10^8^ GC), SARS-CoV-2 (4.5 × 10^5^ GC), AdV (8.7 × 10^5^ GC), RV (1.5 × 10^4^ GC), NV (1.6 × 10^8^ GC) and EnV (2.8 × 10^8^ GC). Because the volume of lake water in contact with the GAC and the adsorption kinetics over time for the viruses are unknown, we cannot translate these findings to an aqueous concentration in the lake. However, understanding the magnitude of viral abundances and therefore fluctuations is noteworthy to understand viral fate and transport in freshwaters. Further, these results provide a basis for comparative analysis against conventional concentration methods, shedding light on their limitations in efficiency in capturing viruses. Table 1 summarizes eight recent studies of viral monitoring in surface and groundwaters globally and includes location, water body type, the volume of water sampled, positive detection rate, maximum concentration (in GC L^−1^, if available), total gene copies (generated for comparison to this study), and method of concentration used.

**Table 1:** Aggregated published data for the detection of pathogenic viruses in freshwater environments.

Region	Water source	Virus	Volume collected and processed (L)	Sample type	% Positive (%)	Maximum gene copies L^-1^	Total gene copies detected	Virus concentration method	References
India	Freshwater Lake	SARS-CoV-2	1	Grab	75	9.9 × 10^4^	9.9 × 10^4^	Ultrafiltration	^55^
Germany	Freshwater Lake	Avian INF	10	Grab	69	N/A	N/A	Ultrafiltration	^53^
Asia	Freshwater Lake	NV	10	Composite	75	4.5 × 10^3^	4.5 × 10^4^	Ultrafiltration	^46^
		AdV			60	5.4 × 10^3^	5.4 × 10^4^		
Asia	Freshwater River	NV GI	1	Grab	13	6.6 × 10^4^	6.6 × 10^4^	Adsorption-elution	^57^
		NV GII			2	6.8 × 10^2^	6.8 × 10^2^		
		AdV			39	3.4 × 10^4^	3.4 × 10^4^		
		Sapovirus			5	1.6 × 10^3^	1.6 × 10^3^		
		Polyomavirus			2	5.0 × 10^2^	5.0 × 10^2^		
		Torque teno virus			3	1.8 × 10^3^	1.8 × 10^3^		
Alberta, Canada	Freshwater River	NV	20	Grab	75	4.2 × 10^0^*	8.4 × 10^1^	Adsorption-elution	^45^*
		RV			100	4.5 × 10^0^*	9.0 × 10^1^		
		Sapovirus			75	4.3 × 10^0^*	8.6 × 10^1^		
		Astrovirus			92	3.8 × 10^0^*	7.6 × 10^1^		
		EnV			58	2.6 × 10^0^*	5.2 × 10^1^		
		AdV			92	4.4 × 10^0^*	8.8 × 10^1^		
		Polyomavirus			83	2.9 × 10^0^*	5.8 × 10^1^		
Mexico	Groundwater	SARS-CoV-2	0.125	Grab	44	3.8 × 10^4^	4.8 × 10^3^	None	^56^
Minnesota, USA	Groundwater	AdV	140-1783	Grab	2.50	6.4 × 10^2^	1.1 × 10^6^	Ultrafiltration	^51^
		EnV			0.90	2.3 × 10^0^	4.1 × 10^3^		
		NV			0.50	2.2 × 10^2^	3.9 × 10^5^		
		RV			1.50	2.3 × 10^2^	4.1 × 10^5^		
Alberta, Canada	Groundwater	AdV	500	Grab	3.00	8.6 × 10^1^	4.3 × 10^4^	Adsorption-elution	^58^
		NV			< 1	N/A	< 60		
		RV			3.00	2.2 × 10^1^	1.1 × 10^4^		
		Polyomavirus			< 1	6.8 × 10^1^	3.4 × 10^4^		
		Reovirus			1.00	3.4 × 10^1^	1.7 × 10^4^		

*Data is presented in terms of median concentration, and only one of six rivers from the study is presented.

When compared to the studies shown in Table 1, the passive sampling method found similar positive detection frequencies of AdV and NV as Pang et al. (2019) and Vergara et al. (2016), with these viruses having the highest detection rates of all viruses studied, in both surface waters and groundwaters^45,46^. Most research on enteric viral presence in freshwater bodies has detected AdV up to 4-log higher concentrations than other enteric viruses^47,48^. The passive sampling results align with past findings of pervasive AdV and a lower abundance of RV. RV abundance has also fluctuated seasonally, with lower abundance observed during warmer months^49,50^. Li et al. (2023) found that RV gene concentrations reduced by over tenfold during summer months compared to winter months^31^. This may account for the low detections of RV in this work, or the virus may not have been widespread during this sampling period. In general, the GAC passive sampler collected orders of magnitude more enteric viral gene targets than other studies in Table 1. A recent groundwater study by Stokdyk et al. (2020) reported very low detection frequencies for all enteric viruses analyzed. However, when adjusted for sampling volume, total gene copies detected approached the order of magnitude observed in the current study^51^. Stokdyk et al. (2020) collected and concentrated upwards of 1800 L of water to quantify viruses of interest. GAC passive sampling may provide a cost-effective and robust method of in-situ viral target concentration for tracking enteric virus presence and abundance in freshwaters.

While comparative research is available for enteric viruses, data on the prevalence of non-enteric viruses in freshwaters, such as SARS-CoV-2, INFA, RSV and MeV, are limited or non-existent. While RSV has been detected in wastewater^40,52^, to our knowledge, this is the first study to evaluate RSV prevalence in a freshwater environment. Likewise, reports on the occurrence of INFA viruses in freshwater environments are still limited. Current research has primarily focused on avian influenza virus subtypes in surface waters, often with low recoverable viral loads^53,54^. Only two previous studies have documented the detection of SARS-CoV-2 in freshwater environments^55,56^. Mahlknechtt et al. (2021) found that SARS-CoV-2 concentrations in surface waters varied based on seasonality and wastewater discharge events, with temporal fluctuations reflecting virus epidemiological trends^56^. Hemalatha et al. (2022) reported no detection of SARS-CoV-2 in peri-urban or rural lakes, while urban lakes exhibited a prevalence of SARS-CoV-2 consistent with clinically reported infections^55^. The GAC passive sampling results show that both RSV and INFA are highly abundant in the study lake, with total gene copies detected in the same range as AdV and NV. Comparative data are scarce for non-enteric viruses in freshwaters; therefore, the results of this study emphasize the need for further research to enhance our understanding of respiratory virus prevalence and behaviour in these environments.

The results from this study show that GAC serves as an effective adsorbent for in situ concentration of viruses in freshwater lakes, either through direct viral adsorption or, more likely, through the adsorption of particle- and sediment-bound viruses. Although the exact mechanisms driving adsorption between GAC and each viral target in these environments are largely still unknown, previous work has observed the ability of GAC to serve as a non-selective media for viral capture in aqueous environments. Cormier et al. (2014) reported that activated carbon could remove upwards of 6 Log PFU^−1^ of MS2 from seeded seawater and freshwater^35^. The capture and recovery of SARS-CoV-2, PMMoV, and CrAssphage have been shown in deionized water and wastewater samples^38^, and RSV and  influenza viruses have been detected using GAC in wastewater^40^. GAC has also been used as an adsorptive media for viral capture in drinking water point-of-use filtration devices, with a known capacity for removing enteric viruses upwards of 99.9%^59^. The results of our work align with these previous reports, showing measurable concentrations of both double-stranded (RV) and single-stranded (EnV, INFA, NV, and SARS-CoV-2) RNA viruses, in enveloped and non-enveloped form, as well as a non-enveloped double-stranded DNA virus (AdV). This study demonstrates the application of passive sampling in freshwater systems to understand viral occurrence.

The present study highlights the range of recoveries for different viral concentration methods and the influence of various sampling conditions. Therefore, factors such as cost, ease of use, need for recovery controls, and a method's ability to achieve the study's specific objectives should be considered when selecting a method^60^. This approach can lead to more practical and adequate decision-making in monitoring viral contamination in various environmental samples. To ensure the safety of recreational and drinking water supplies, it is crucial to consider the limitations of grab samples and explore alternative methods, such as passive samplers, for monitoring viral occurrence in freshwater environments.

## Summary of future research needs

Information on the concentration of viruses in natural waters is critical to understanding the risk of infection and the effectiveness of controls to limit exposure. However, current knowledge on the occurrence of viruses in freshwater environments is largely limited to enteroviruses, and much of this work is constrained to academic studies and occasional commercial research, with an overreliance on fecal indicator organisms for policy development, water treatment standards and public health guidelines. There is a limited understanding of the spatial and hydrological influence of viral abundances in freshwater, regardless of the concentration method used. Conventional concentration methods often rely on water collected at a single point in time and from a single location. Passive sampling methods offer valuable insights into time-integrated viral concentrations, offering a more accurate spatial and temporal representation of viral abundance in water sources. The emerging information obtained through passive sampling is currently of great interest and has generated ongoing discussions in the research field^43^. Future research should work to enhance our understanding of viral dynamics throughout the deployment phase and also establish a baseline for evaluating the efficiency of passive samplers in direct relation to volume-based metrics, providing valuable insights for future monitoring and management efforts.

Applying GAC-based passive sampling could advance viral monitoring in recreational and drinking water sources to inform water quality management better. To fully leverage the utility of passive sampling for viral monitoring, future studies need to investigate temporal and spatial dimensions of passive sampling in freshwater bodies. To establish effective environmental surveillance and guide policy on viral monitoring, future work must establish passive sampling procedures for fecal contamination and viral abundance in freshwater environments. This can inform public health decisions and refine drinking water treatment technologies.

### Conclusions

Despite advances in drinking water and wastewater treatment, water-related pathogenic viruses remain a public health concern globally. We have presented GAC passive sampling as a potentially viable, simple, and cost-effective way of simultaneous in situ concentration of a range of enteric and non-enteric viruses in freshwaters. Future work is needed to characterize better adsorptive mechanisms, the role of equilibrium and kinetics in viral or soil-bound viral uptake, survival and transport, and the relationship between passive sampling and viral loads and exposure risks to the public. Our findings may have far-reaching implications for reducing barriers associated with viral monitoring across various environmental contexts.

## Materials and methods

### Sampling location and sample collection methods

To study the occurrence of the selected viruses in a recreational freshwater lake, samples were collected from two locations in June, July and August of 2022. The study lake is located in a populated urban area of Nova Scotia. The lake is ~ 1.3 km long, 500 m wide, and 11 m deep and is surrounded by mixed residential/commercial properties, recreational facilities (canoe/kayak clubs) and several roadways. There are no known wastewater inputs to the lake other than potential recreational swimming or potential fecal material from wild and domestic animals. Two sampling locations were monitored to evaluate the viral abundance throughout the lake. At the first site (Site 1), passive samplers were deployed from a floating dock adjacent to a popular recreational beach, with the passive sampler suspended using nylon rope approximately 1.5 m below the surface. For the second site (Site 2), the passive sampler was secured to the shoreline again using nylon rope and deployed approximately 3 m from the water’s edge; this sampler rested on the bottom of the lake (immediately adjacent to the sediment layer) at a depth of approximately 1–2 m below the surface. The passive sampler was engineered with a density that naturally facilitated its submersion, eliminating the need for supplementary weights for stable suspension in the lake. Generally, Site 2 had less recreational activity than Site 1.

Passive sampling was conducted using an adapted version of the 3D-printed passive sampler developed by Hayes et al. 2021^61^. For each deployment, 3 g of GAC was placed in a heat-sealable nylon mesh sleeve with ~ 25-μm pores and was put in the passive sampler to capture viral targets^38^. Passive samplers were deployed for one week, a duration found to optimally balance effective analyte adsorption with the GAC adsorption capacity^38^. Following week long deployments, samplers were collected and placed in sealable plastic bags for transport to the lab and a new sampler was deployed for the subsequent weeks sampling. Simultaneously to the deployment and retrieval of the passive samplers, grab samples were collected from the exact locations at approximately the same depth as the passive samples. Grab samples used for nucleic acid extraction were collected in sterilized 500 mL Nalgene bottles, and those used for water quality characterization were collected in acid (5% HCL) washed 1 L Nalgene bottles. These volumes were chosen based on Health Canada’s sampling recommendations of between 200 and 500 mL for FIB analysis in recreational waters^21^. This recreational water guidance does not specify recommendations for viral monitoring protocols. Passive and grab samples were placed in coolers packed with ice until they were delivered to the lab, where they were stored at 4 °C. In total, 20 passive samples and 33 grab samples were collected across both Site 1 and Site 2 during the three month sampling period.

### Sample processing

#### GAC passive sampling

Viral RNA was desorbed from GAC using a modified elution protocol adapted from Hayes et al. 2022^38^. Briefly, GAC was removed from the passive sampler and eluted with 6 mL of a Tween20®-based buffer solution; a 1 mL aliquot of the eluate was then placed in a bead beating tube containing 500 μL of lysis buffer (BioGX, Birmingham, AL, USA). The resulting lysate was transferred to a sterile Eppendorf tube and stored at − 20 °C while awaiting RT-qPCR analysis.

#### Grab sampling

Grab samples were processed by concentrating a ~ 100 mL aliquot of the 500 mL sample on a 0.8 μm acrylic copolymer filter membrane (Cole-Parmer, Vernon Hills, IL, USA) using a sterile syringe filter. Using sterile tweezers, the filter membrane was placed in a bead-beating tube containing 500 μL of lysis buffer (BioGX, Birmingham, Alabama, USA). The resulting lysate was transferred to a sterile Eppendorf tube and stored at − 20 °C while awaiting RT-qPCR analysis.

### Nucleic acid extraction

Nucleic acids were extracted from passive and grab samples within 24-h of sample collection and then stored at − 80 °C until subsequent RT-qPCR analysis. To minimize contamination during nucleic acid extraction and RT-qPCR preparation, a Thermo Scientific 1300 Series A2 biosafety cabinet (Thermo Fisher Scientific, Oakwood, OH, USA) and a Mystaire MY Model PCR Prep Station Class 100 laminar flow enclosure were utilized, respectively.

### RT-qPCR reaction and thermocycling parameters

The isolated RNA/DNA was utilized for viral detection of SARS-CoV-2, INFA, RSV-A, MeV, EnV, RV, and NV through RT-qPCR techniques. Primer, probe sequences, working concentrations, and the thermocycling conditions used for each viral target are listed in Table S2. Primers and probes for each assay were produced by Integrated DNA Technologies (IDT; Coralville, IA, USA). RT-qPCR reactions comprised 20 μL mixtures, consisting of 3 μL of isolated nucleic acid and 5 μL of TaqMan Fast Virus 1-Step Multiplex Master Mix (ThermoFisher, Tewksbury, MA, US). Samples were analyzed using the Gene Count Q-96 thermocycler instrument (LuminUltra Technologies, Ltd., Fredericton, NB, CA).

### Water quality analysis

In-situ pH, dissolved oxygen, conductivity, total dissolved solids, and temperature were measured using a YSI Professional Plus sonde. All laboratory water quality characterization of grab samples was completed within 48-h of sample collection. Concentrations of dissolved and total organic carbon (DOC, TOC) were quantified using a Total Organic Carbon Analyser (Shimadzu, TOC-VCPH). Turbidity was measured using a HACH 2100AN 32 turbidimeter. Ultraviolet absorbance at 254 nm (UV_254_) and actual colour were measured on a HACH Spectrophotometer. Total aluminum, iron, and phosphorus were analyzed via inductively coupled plasma-mass spectrometry (ICP-MS) using an X-Series II ICP-MS (Thermo Fisher Scientific, Oakwood, OH, USA).

### Statistical analysis

Concentrations of viral target detections between the two sampling sites were compared using the paired samples Wilcoxon test with a significance level of α = 0.05. All statistical analyses and generation of figures were completed using R Studio (version 4.2.3) and packages including tidyverse, scales, janitor and ggtext^62–65^. A corresponding Cq value characterized all samples, and gene concentrations were calculated based on the respective calibration curves generated for each viral target (Table S3). To determine the performance of each assay, the slopes (S) of the regression lines were used to calculate the amplification efficiency (ε) of each calibration curve, according to the formula ε = 10^|−1/s|^ − 1. Total gene copies (GC) recovered in passive and grab samples were computed by Eqs. (1).

1 $$Total\,GC=\frac{\frac{GC}{rxn}*\frac{rxn}{3 \mathrm{\mu L}}*(500\,\mathrm{\mu L}+1000\,\mathrm{\mu L})}{1000\,\mathrm{\mu L}}*(6000\,\mu L)$$

### Quality assurance-quality control (QA-QC)

Standards outlined in the minimum information for publication of quantitative real-time PCR experiments (MIQE) guidelines (Table S4) and environmental microbiology minimum information (EMMI) guidelines were consulted to ensure the reliability of the RT-qPCR results^66,67^. All consumables were either purchased pre-sterilized or were autoclave sterilized. All RT-qPCR reagents were prepared as single-use aliquots to ensure the reliability of RT-qPCR results and prevent potential issues such as cross-contamination or degradation of stock solutions. The quality and functionality of a freshly acquired batch of RT-qPCR reagents, including primers, probes, and mastermix, were verified before utilization. This verification process involved analyzing ten replicates of no-template controls alongside at least one positive template control^68^. Each workstation included its own laboratory equipment, along with all laboratory supplies, reagents, and personal protective equipment. Ultraviolet light (90-min exposure) and DNase/RNase-free water were used on all lab work surfaces after decontamination by 1% bleach for ~ 30 min. To ensure methodological integrity, unidirectional workflow was implemented, accompanied by the establishment of distinct autonomous working areas. For nucleic acid extractions, a Thermo Scientific 1300 Series A2 biosafety cabinet was used, and for qPCR reaction prep a Mystaire MY Model PCR Prep Station Class 100 laminar flow enclosure was used. Several controls were used through each sample processing and analysis procedures, including a concentration control to monitor the process efficiency of each analysis (bacteriophage MS2), a negative nucleic acid extraction control, and positive and negative RT-qPCR template controls. DNase/RNase-free water served as the process and template negative controls, and synthetic reference material for each virus was used for positive controls during RT-qPCR analysis (Table S5). Quantitative results were reported based on a Cq value threshold of ≤ 37 cycles. Results below this threshold were considered non-detect. Any results obtained from samples where process blanks or no template controls were amplified, were excluded from the analysis and rerun.

### Supplementary Information

Supplementary Information.

## Data Availability

All data and code needed to reproduce the analyses are available online (https://github.com/djredden/lake_viruses/tree/main).
