# Supplementary material for: Enhanced detection of viruses for improved water safety
Source: Sci Rep. 2023 Oct 13;13:17336. doi: 10.1038/s41598-023-44528-2 (PMC10575868; doi:10.1038/s41598-023-44528-2)
Supplement: Supplementary file 1 — Supplementary Information. [file 41598_2023_44528_MOESM1_ESM.pdf]

Enhanced detection of viruses for improved water safety

Emalie K. Hayes[1], Madison T. Gouthro[1], Megan Fuller[1], David J. Redden[1] and Graham A. Gagnon[1*]
[1]Centre for Water Resources Studies, Department of Civil & Resource Engineering, Dalhousie University, 1360 Barrington Street, Halifax, Nova Scotia B3H 4R2, Canada

* Corresponding author

Dr. Graham Gagnon

E-mail address: Graham.gagnon@dal.ca

Table S1. Median values and standard deviations for select water chemistry parameters measured between May and November 2022 at the two sample locations in Lake Banook.

	Units	Median	Standard Deviation
Total Aluminum	µg L^{-1}	7.04	3.05
Colour	Pt-Co	10	11.15
Conductivity	µs cm^{-1}	844	91.97
Dissolved oxygen	mg L^{-1}	7.65	1.16
Dissolved organic carbon	mg L^{-1}	2.21	0.1
Total Iron	µg L^{-1}	37.65	16.17
Total dissolved solids	mg L^{-1}	597.5	38.98
Total organic carbon	mg L^{-1}	2.21	0.08
Temperature	°C	19.75	3.49
Total P	µg L^{-1}	8.64	8.31
Turbidity	NTU	0.76	0.74
UV_{254}	cm^{-1}	0.05	0.01
pH	-	7.5	0.38

Table S2. Oligonucleotide sequences for the respective primers, and probes used for each virus. The working concentrations for each qPCR assay. Oligonucleotide probe sequences were labelled at the 5′-end with fluorescent reporter dyes and quenched with a Blackhole Quencher 1 or 2 at the 3′-end.

Targets	Genes	Sequences (5'–3')	Concentration (nM)	Amplicon size (bp)	Cycling Conditions	Ref.
INFA[1]	Matrix protein (M1)	**F1:**CAAGACCAATCYTGTCACCTCTGAC	400<br>600<br>400<br>200<br>200	106	2 min at 25°C, 15 min at 50°C, 2 mins at 95°C, 45 cycles of 15 s at 95°C, and 30 s at 60°C	[1]
		**R1:**GCATTYTGGACAAAVCGTCTACG				
		**F2:**CAAGACCAATYCTGTCACCTYTGAC				
		**R2:**GCATTTTGGATAAAGCGTCTACG				

		**P:**TGCAGTCCTCGCTCACTGGGCACG				
SARS-CoV-2[1]	Nucleocapsid (N1)	**F:**CTGCAGATTTGGATGATTTCTCC<br>**R:**CCTTGTGTGGTCTGCATGAGTTTAG<br>**P:**ATTGCAACAATCCATGAGCAGTGCTGACTC	100<br>200<br>200	92		
RSV-A[1]	Nucleoprotein (N)	**F:**GCTCTTAGCAAAGTCAAGTTGAATGA<br>**R:**TGCTCCGTTGGATGGTGTATT<br>**P:**ACACTCAACAAAGATCAACTTCTGTCATCCAGC	500<br>500<br>200	82		
MeV[1]	Nucleoprotein (N)	**F:**ATATATCGTAGAGGCAGGATTAG<br>**R:**AGGACTCAAGTGTGGATAAC<br>**P:**AAACTATGTATCCTGCTCTTGG	500<br>500<br>200	119		
EnV	Polyprotein (PP)	**F:**GATTGTCACCATAAGCAGC<br>**R:**GCCCTGAATGCGGCTAATC<br>**P:**CGGAACCGACTACTTTGGGTGTCCGT	400<br>400<br>100	148	5 min at 50°C, 20s at 95°C, 40 cycles of 3 s at 95°C, and 30 s at 60°C	[2]
AdV[2]	Hexon structure gene of species F (type 40/41)	**F:**TCCGACCCACGATGTAACCA<br>**R:**CACGGCCAGCGTAAAGCG<br>**P:**ACAGGTCACAGCGACT	250<br>250<br>100	112		[3]
RV[2]	non-structural protein 3 (NSP3) gene) species A	**F1:** ACCATCTACACATGACCCTCTATG<br>**F2:** ACCATCTTCACGTAACCCTCTATG<br>**R:** ACATAACGCCCCTATAGCCATTT<br>**P:**AATAGTTAAAAGCTAACACTGTC	250<br>250<br>100	83		
NV[2]	ORF2 (the junction of polymerase and capsid gene) of the genotype II	**F:**CCAATGTTCAGATGGATGAGATTCTC<br>**R:**TCGACGCCATCTTCATTCACA<br>**P:**ATCGCCCTCCCACGT	250<br>250<br>100	92		

MS2	Maturation & structural protein (*mat* and *cp* genes)	**F:**GTCCATACCTTAGATGCGTTAGC<br>**R:**CCGTTAGCGAAGTTGCTTGG<br>**P:**ACGTCGCCAGTTCCGCCATTGTCG	400<br>400<br>250	160	30 min at 55°C, 3 min at 94°C, followed by 45 cycles 15 s at 94°C and 1 min at 60°C	[4]

[1] Multiplex RT-qPCR assay previously validated in the work of Hayes et al., (2023) for the simultaneous detection of SARS-CoV-2, INFA, RSV, and MeV [1]. [2] Multiplex qPCR assay for the detection of human rotavirus, enteric adenovirus, and human norovirus, formerly validated by [3].

Table S3. Viral calibration curve information for each viral target.

**INFA**		**SARS-CoV-2**		**MeV**		**RSV**	
R2	0.95	R2	0.96	R2	0.97	R2	0.96
y-int	38.78	y-int	40.71	y-int	41.16	y-int	38.97
Slope	-3.17	Slope	-3.76	Slope	-3.84	Slope	-3.53
Efficiency (%)	106.71	Efficiency (%)	84.48	Efficiency (%)	82.14	Efficiency (%)	91.90
**EnV**		**Norovirus**		**Adenovirus**		**Rotavirus**	
R2	0.99	R2	0.99	R2	0.99	R2	0.99
y-int	37.48	y-int	40.34	y-int	39.22	y-int	40.32
Slope	-3.13	Slope	-3.38	Slope	-3.28	Slope	-3.50
Efficiency (%)	108.37	Efficiency (%)	97.66	Efficiency (%)	101.5	Efficiency (%)	92.8

Table S4. The Minimal Information for Publications on Quantitative Real-Time PCR Experiments (MIQE) checklist of essential and desirable information that should be reported to enable the reviewer to judge the validity of the paper and the reader to repeat the experiment and reproduce the results.

**Category**	**Item**	**Paper Location**	**Author Comments**	**Checklist**
**Sample**	Type	Methods	Passive and grab samples	✓
	Method of dissection/procurement	Methods and Supplemental	No dissection, synthetic template manufacturers, and sample collection are described	✓
	Processing procedure	Methods	Pre-RNA extraction processing	✓
	If frozen, how and how quickly?	Methods		✓
	If fixed, with what and how quickly?	N/A	No fixing performed (i.e., no formalin-fixed procedures)	N/A
	Storage conditions and duration	Methods and Supplemental	Template control storage conditions are described in SI	✓

			based on manufacturers recommendations.	
**Extraction**	Method or instrument	Methods	Commercial kit	✓
	Reagents/kits/modifications	Methods		✓
	DNAse or RNAse treatment	N/A	No specific treatment completed; purification steps included in commercial kit used (LuminUltra Technologies Ltd).	N/A
	Evidence for lack of contamination (DNA or RNA)	N/A	RNA extract dilution during RT-qPCR analysis	X
	Nucleic acid quantification	Supplemental	RT-qPCR analysis, and multiplex RT-qPCR analysis	✓
	RNA integrity	Supplemental		✓
**Reverse transcription**	Complete reaction conditions, including all components and their concentrations	Supplemental		✓
	RNA amount and reaction volume	Methods		✓
	Priming oligo sequence(s)	Supplemental		✓
	Cq values with and without reverse transcriptase	N/A	One-step RT-qPCR was used in this study	✓
**qPCR target**	Sequence accession number	Supplemental	Included in SI / methods for primer sequences and template sequences used	✓
	Amplicon length	Supplemental		✓
	In silico specificity (BLAST)	Validated in previous publications		✓
	Location by exon/intron	Supplemental		✓
	Identify the splice variants amplified	N/A		×
	All primer/probe sequences	Supplemental		✓
	Location and identity of any oligonucleotide modifications	Supplemental		✓

	Complete reaction conditions, including all components and their concentrations	Methods & Supplemental		✓
**qPCR protocol**	cDNA/DNA amount and reaction volume	Methods		✓
	Instrument identification and complete thermocycling parameters	Methods & Supplemental		✓
	Evidence for PCR specificity (gels, sequencing, or melting curves)	Validated in previous publications		✓
**qPCR validation**	Template inhibition data (template titrations)	Supplemental	Extract dilutions were evaluated for inhibition, no inhibition was expected.	✓
	For SYBR Green I reactions, the Cq of the no template control	Methods	Didn't include data where no template control failed	✓
	Calibration curves with slope and intercept	Supplemental/previously validated in other published work		✓
	PCR efficiency from the slope	Supplemental		✓
	r^2 of the calibration curve	Supplemental		✓
	Evidence for the linear dynamic range	Supplemental		✓
	Evidence for the limit of detection	Validated in previous publications		✓
	For multiplexed assays, the efficiency and limit of detection of each assay	Supplemental		✓
	qPCR analysis method/software	Supplemental & Methods		✓
**Data analysis**	Method of Cq determination	Methods		✓
	Results of no template controls	Methods		✓
	Justification of number and choice of reference genes	Supplemental		✓

	Normalization method			X
	Number and stage (reverse transcription or qPCR) of technical replicates	Supplemental		✓
	Intra-assay variation in terms of concentration, not Cq	Validated in previous publications		✓
	Statistical methods/software	Methods		✓

Table S5. Synthetic viral RNA reference material used in verification and validation of RT-qPCR assays.

Company	Name	Accession # / Designated Strain	Manufacturer Specifications	Storage Conditions
Twist Bioscience	Influenza A H1N1 (2009)	NC_20643<br>NC_026431<br>NC_026432<br>NC_026433<br>NC_026434<br>NC_026435<br>NC_026436<br>NC_026437<br>NC_026438	~1×10^6 copies/µL	-90 ºC to -70ºC
	Measles virus	NC_001498.1		
	SARS-CoV-2 Control 48	Omicron B.1.1.529, BA.1 lineage<br><br>**GISAID ID:** EEPI_ISL_6841980<br>**GISAID NAME:** Hong Kong/HKU-211129-001/2021		
ATCC®	Human respiratory syncytial virus (RSV) A2	(ATCC® VR-1540DQ™) / MW582527.1	~1 x 10^6 copies/µL	-80ºC
Integrated DNA Technologies gBlocks, Gene Fragments	Bacteriophage MS2	NC_001417	~1×10^{13} copies/µL	-20ºC
	Norovirus	X86557	10 ng/uL	

	Adenovirus	D13781		
	Rotavirus	X81436		
	Enterovirus	MW473684.1		

Supplementary References

[1] Hayes EK, Gouthro MT, LeBlanc JJ, Gagnon GA. Simultaneous detection of SARS-CoV-2, influenza A, respiratory syncytial virus, and measles in wastewater by multiplex RT-qPCR. Science of The Total Environment 2023:164261. https://doi.org/10.1016/j.scitotenv.2023.164261.

[2] Coudray-Meunier C, Fraisse A, Martin-Latil S, Delannoy S, Fach P, Perelle S. A Novel High-Throughput Method for Molecular Detection of Human Pathogenic Viruses Using a Nanofluidic Real-Time PCR System. PLOS ONE 2016;11:e0147832. https://doi.org/10.1371/journal.pone.0147832.

[3] Lee D-Y, Leung K, Lee H, Habash M. Simultaneous Detection of Selected Enteric Viruses in Water Samples by Multiplex Quantitative PCR | SpringerLink. Water Air Soil Pollut 2016;227. https://doi.org/10.1007/s11270-016-2811-5.

[4] Gendron L, Verreault D, Veillette M, Moineau S, Duchaine C. Evaluation of Filters for the Sampling and Quantification of RNA Phage Aerosols. Aerosol Science and Technology 2010;44:893–901. https://doi.org/10.1080/02786826.2010.501351.
